# Effectiveness of Wearable Devices for Diabetes Management: An Overview of Systematic Reviews and Meta‐Analyses

**DOI:** 10.1155/jdr/1464982

**Published:** 2026-04-09

**Authors:** Muxiarepu Talaiti, Xiaoqin Liao, Nuerguli Abulimiti, Meihua Zeng, Yangyang Wang

**Affiliations:** ^1^ School of Nursing, Shanghai University of Traditional Chinese Medicine, Shanghai, China, shutcm.edu.cn

## Abstract

**Objective:**

Wearable devices play an important role in managing diabetic patients, and patient demand for them is also on the rise. This overview systematically summarizes and appraises the current evidence on wearable device–based interventions for diabetic patients.

**Methods:**

Systematic reviews/meta‐analyses on wearable device interventions for diabetic patients were searched in 10 databases from inception to February 21, 2025. Two researchers independently screened and extracted the data. Methodological quality was assessed using the AMSTAR‐2 tool, report quality via the PRISMA 2020 statement, and evidence quality through the GRADE approach.

**Results:**

Thirty‐one studies were included. PRISMA 2020 reporting quality scores ranged from 20 to 26.5 points. Regarding AMSTAR‐2 methodological quality, 25.8% (8/31) were rated high, 38.7% (12/31) low, and 35.5% (11/31) very low. GRADE assessed 97 bodies of evidence (four outcome categories, 27 specific indicators), with only 1 (1.03%) rated as high quality. Wearable devices for diabetes management primarily fall into four categories: blood glucose monitoring, therapeutic interventions, exercise interventions, and comprehensive management systems. Blood glucose monitoring devices were the most widely used and were associated with significant increases in time in range (TIR) and reductions in glycated hemoglobin (HbA1c). Among therapeutic devices, specialized footwear reduced the risk of diabetic foot ulcers, whereas exercise interventions increased daily step counts.

**Conclusion:**

Wearable devices may benefit glycemic control, exercise management, and foot ulcer prevention in diabetic patients, but evidence for preventing diabetic ketoacidosis and improving psychological outcomes remains limited. These findings should be interpreted cautiously due to the generally low quality of existing evidence. High‐quality, long‐term studies are needed.

## 1. Introduction

As a globally prevalent chronic disease, diabetes has experienced a sustained and accelerating increase in incidence, becoming a significant public health and socioeconomic burden across diverse populations and healthcare systems [1]. Owing to its chronic and progressive nature, effective management fundamentally centers on maintaining stable blood glucose levels to prevent or delay the onset of severe microvascular and macrovascular complications, including cardiovascular disease, renal failure, and retinopathy [2]. To achieve long‐term glycemic stability, patients are required to undertake complex and frequent self‐management tasks such as blood glucose monitoring, insulin administration, dietary regulation, and physical activity planning [3]. However, these repetitive and demanding behaviors often contribute to reduced treatment adherence, psychological fatigue, and compromised self‐efficacy, ultimately affecting glycemic outcomes [4].

In recent years, rapid advances in wearable technology have introduced novel and promising pathways for diabetes management [5]. Monitoring‐oriented wearable devices—such as continuous glucose monitoring (CGM) systems—enable real‐time, 24‐h dynamic tracking of glucose fluctuations, significantly reducing dependence on conventional finger‐stick sampling and enhancing the precision of glycemic assessment [6]. Therapeutic wearable devices, including closed‐loop or artificial pancreas systems, further integrate sensing and insulin‐delivery capabilities, allowing automated or semiautomated insulin titration to optimize postprandial glycemic control and mitigate acute glycemic events [7]. Additionally, lifestyle‐focused wearables (e.g., pedometers and smart bands) generate objective physical activity metrics, supporting personalized exercise prescription and promoting behavioral engagement [8]. Despite an expanding body of systematic reviews and meta‐analyses examining the application of wearable devices in diabetes management, the current evidence base remains undermined by heterogeneity in study design, variability in outcome measures, and inconsistencies in methodological quality. These limitations constrain the reliability and generalizability of existing findings. This study adopts a systematic reevaluation approach to comprehensively appraise the methodological quality and evidentiary strength of published research, thereby delineating the clinical efficacy boundaries of wearable device‐based interventions and providing evidence‐informed guidance for future clinical practice and research development.

## 2. Methods

### 2.1. Protocol and Registration

The research protocol was registered in the International Prospective Systematic Review Registry (PROSPERO) on February 14, 2025, under the Registration Number CRD42025646643.

### 2.2. Eligibility Criteria

#### 2.2.1. Literature Inclusion Criteria

According to the PICOS principle [9], the inclusion criteria were as follows: (1) population (P)—nonpregnant diabetes patients. (2) Intervention (I)—use of wearable devices by diabetes patients for blood glucose monitoring, insulin therapy, and so on. Wearable devices refer to portable electronic equipment incorporating smart sensors, worn by users to collect physiological parameters and signals, and exchange data with computer terminals [10]. Wearable devices applied in diabetes management encompass glucose monitoring devices (patches, wristbands, etc.), therapeutic devices (insulin pumps), and management devices (activity trackers and glucose alerts). (3) Control measures (C)—conventional care or placebo control; may also involve comparisons between different wearable devices. (4) Outcome measures (O)—primary measures focus on blood glucose‐related indicators; secondary measures include complications, weight/exercise metrics, psychological state indicators, and so on. (5) Study design (S)—systematic reviews or meta‐analyses based on randomized controlled trials (RCTs).

#### 2.2.2. Exclusion Criteria

Studies were excluded if they (1) were protocols for systematic reviews or meta‐analyses; (2) were duplicate publications; (3) were studies for which full text could not be obtained; (4) were studies with critical methodological flaws, incomplete essential data, or unclear outcome assessments.

### 2.3. Data Sources and Search Strategy

The literatures were retrieved from PubMed, Embase, Cochrane, Web of Science, EBSCO, Scopus, SinoMed, CNKI, Wanfang and VIP on February 21, 2025. We adopted a strategy that combines keyword search with free word search, and the keywords include “Diabetes Mellitus,” “Wearable Electronic Devices,” “systematic review,” “meta‐analysis.” The literature search strategy of the PubMed database is shown in Table 1, which was reasonably tuned for each database. Supplementary manual citation tracking ensured comprehensive coverage.

**Table 1:** Search strategy for the PubMed database.

Query	Search term
#1	(((((((((((((((((((((wearable technology[Title/Abstract]) OR (wearable sensor[Title/Abstract])) OR (wearable biosensors[Title/Abstract])) OR (ambulatory monitoring[Title/Abstract])) OR (microchip[Title/Abstract])) OR (electronic skin[Title/Abstract])) OR (watch[Title/Abstract])) OR (wristbands[Title/Abstract])) OR (pulse oximeter[Title/Abstract])) OR (finger pulse[Title/Abstract])) OR (patches[Title/Abstract])) OR (belts[Title/Abstract])) OR (glasses[Title/Abstract])) OR (continuous glucose monitoring[Title/Abstract])) OR (helmets[Title/Abstract])) OR (bracelet[Title/Abstract])) OR (shoes[Title/Abstract])) OR (smart shoes[Title/Abstract])) OR (insoles[Title/Abstract])) OR (socks[Title/Abstract])) OR (accelerometry[Title/Abstract])) OR (pedomet[Title/Abstract])
#2	“diabetes mellitus”[MeSH Terms] OR “diabetes insipidus”[MeSH Terms] OR “diabetes “[MeSH Terms] OR (“diabetes”[Title/Abstract] OR “diabetes mellitus”[Title/Abstract])
#3	((systematic review [Title/Abstract]) OR (meta analysis Title/Abstract])) OR (meta [Title/Abstract])
#4	#1 AND #2 AND #3

### 2.4. Eligibility Assessment and Data Extraction

The literature screening (MT and MH‐Z) and information extraction (NA and YY‐W) were performed independently by two researchers. EndNote 21 was used to remove duplicate records. First, a preliminary screening was conducted by reviewing the titles and abstracts, followed by full‐text screening to identify studies that met the inclusion criteria. From the included studies, the following data were extracted: publication year, authors, number and sample size of included RCTs, type of diabetes, type of wearable device, interventions for the experimental and control groups, outcome measures, and the tools used for methodological and evidence quality assessment. The disagreement between the two researchers was resolved by a third researcher.

### 2.5. Quality Assessment

Two researchers (MT and NA) independently assessed the methodological and evidentiary quality of the included SRs/MAs.

### 2.6. Assessment of Methodological Quality

The reporting quality of each review was assessed using the PRISMA 2020 statement [11], which includes 27 items. Each item was scored as 1 point for complete reporting, 0.5 points for partial reporting, and 0 points for nonreporting, yielding a maximum score of 27. A total score of ≤ 15 was considered indicative of severe reporting deficiencies, scores of 16–21 reflected moderate deficiencies, and scores of 22–27 denoted relatively complete reporting. Methodological quality was evaluated using the AMSTAR 2 tool [12, 13], which comprises 16 items including seven critical domains, classifying reviews into four quality levels (high, moderate, low, and critically low). Notably, failure to meet any critical domain resulted in an automatic downgrade to low or critically low quality. Evidence certainty was further appraised using the GRADE approach [14] across five domains—risk of bias, inconsistency (*I* ^2^), indirectness, imprecision (sample size), and publication bias—resulting in four levels of evidence quality (high, moderate, low, and very low). Only outcomes for which meta‐analytic effect estimates were available were evaluated; individual RCTs without pooled effects were not independently graded.

## 3. Result

### 3.1. Results on Literature Search and Selection

**Figure 1:**
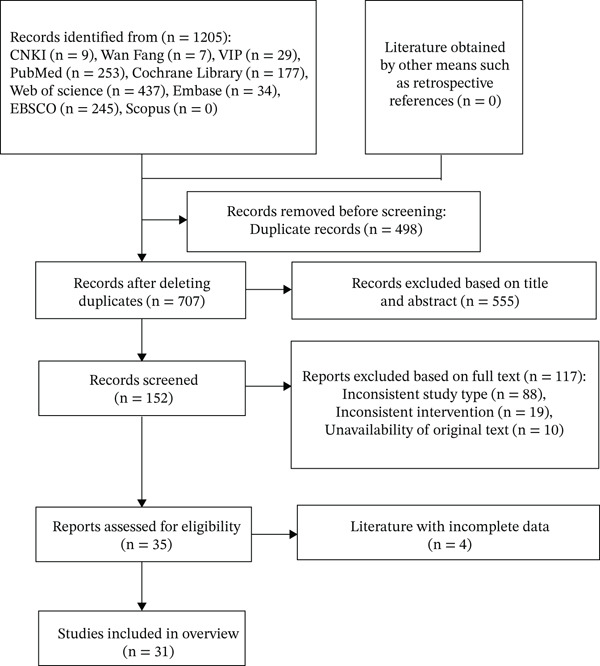
The flowchart of the screening process.

A systematic search of 10 databases yielded 1205 initial records. Following duplicate record removal (*n* = 498), 707 publications proceeded to the title/abstract screening stage, with 152 advancing to full‐text assessment. After reviewing, 117 were excluded because they were not consistent with our research content, and four were excluded due to incomplete data. Finally, 31 studies were discussed. The literature screening process and the results are shown in Figure 1.

### 3.2. Description of Included SRs/MAs

The characteristics of the included SRs/MAs are presented in Table 2. These reviews were all published between 2008 and 2024, 29 of which were in English, and the remaining two were in Chinese, with 22 (71.0%) published after 2019, indicating a surge in research interest in recent years. All SRs/MAs incorporated RCT evidence, with the number of included trials ranging from 5 to 26 and sample sizes ranging from 335 to 2879 participants, the follow‐up time ranging from 3 days to 2 years. The study populations covered Type 1 diabetes (12 reviews), Type 2 diabetes (T2D, 13 reviews), and mixed cohorts including both T1D and T2D (6 reviews). The included reviews examined multiple categories of wearable devices and correspondingly reported a broader range of outcome indicators. Regarding RCT quality assessment, 27 reviews used the Cochrane risk‐of‐bias tool, two reviews used the Jadad scale, one review used the Joanna Briggs Institute Critical Appraisal Tools, and one review adopted the Maastricht–Amsterdam Consensus List. Twelve reviews conducted GRADE evidence assessments.

**Table 2:** Characteristics of the included SRs/MAs (*n* = 31).

No.	Citation	Trials (subjects)	Participants’ diagnosis	Types of wearable devices	Intervention group	Control group	Outcomes	Methodological quality assessment	Evidence quality assessment
1	2008Chetty[15]	7(335)	T1D	Continuous Glucose Monitoring System (Subcutaneous Sensor)	CGM	SMBG	(1)	Jadad scale	NR
2	2011Pickup[16]	6(892)	T1D	Continuous Glucose Monitoring System (Subcutaneous Sensor)	RT‐CGM	SMBG	(1)(35)	NR	NR
3	2012Szypowska[17]	7(948)	T1D	Continuous Glucose Monitoring System (Subcutaneous Sensor)	RT‐CGM	SMBG	(1)(2)(3)(4)(5)(6)(7)	Cochrane criteria	NR
4	2013Poolsup[18]	14(1025)	T1D children (≤18 years) and T2D adults (≥18 years)	Continuous Glucose Monitoring System (Subcutaneous Sensor)	CGM	SMBG	(1)	Maastricht Amsterdam scale	NR
5	2017Benkhadra [19]	11(1530)	T1D	Continuous Glucose Monitoring System (Subcutaneous Sensor)	RT‐CGM	usual care	(1)(2)	Cochrane criteria	NR
6	2019Ida[20]	7(669)	T2D	Continuous Glucose Monitoring System (Subcutaneous Sensor)	CGM	SMBG	(1)(7)(8)(10) (15)(26)(27)	Cochrane criteria	NR
7	2020Cowart[21]	9(635)	T1D \T2D	Continuous Glucose Monitoring System (Subcutaneous Sensor)	isCGM	SMBG	(9)(10)(12) (15)	Cochrane criteria	NR
8	2022Elbalshy[22]	22(2879)	T1D	Continuous Glucose Monitoring System (Subcutaneous Sensor)	Adjunctive CGM、Non‐Adjunctive CGM、isCGM	SMBG	(1)(9)(10)(11)(12)	Cochrane criteria	NR
9	2022Teo[23]	22(2188)	community‐dwelling patients with T1D	Continuous Glucose Monitoring System (Subcutaneous Sensor)	CGM	SMBG	(1)(5)(22)	Cochrane criteria	GRADE framework
10	2022Wang[24]	11(1745)	T1D \T2D	Continuous Glucose Monitoring System (Subcutaneous Sensor)	CGM	SMBG	(1)(9)(10)(11)(16)	Cochrane criteria	NR
11	2024Ferreira[25]	6(407)	T2D patients on non‐insulin therapy	Continuous Glucose Monitoring System (Subcutaneous Sensor)	CGM	SMBG	(9)(10)(11)(12)(14)(15) (16)	Cochrane criteria	GRADE framework
12	2024Kong[26]	17(1619)	T2D adults (≥18 years)	Continuous Glucose Monitoring System (Subcutaneous Sensor)	CGM	SMBG	(1)(7)(8)(9)(10)(11)(15)(16)(20)(23)(30)(31)	JBI Critical Appraisal criteria	NR
13	2024Jancev[27]	12(1248)	T2D	Continuous Glucose Monitoring System (Subcutaneous Sensor)	CGM	SMBG	(1)(2)(9)(10) (11)(12)(13)	Cochrane criteria	GRADE framework
14	2024Lu[28]	11(1425)	T2D	Continuous Glucose Monitoring System (Subcutaneous Sensor)	CGM	SMBG	(1)(9)(10)(11)	Cochrane criteria	NR
15	2024Seidu[29]	26(2783)	T2D	Continuous Glucose Monitoring System (Subcutaneous Sensor)	CGM、isCGM	SMBG	(1)(6)(8)(9)(10)(11)(12)(15)(23)(26)(27)(31)(34)	Cochrane criteria	GRADE framework
16	2024Zhou[30]	5(446)	T1D	Continuous Glucose Monitoring System (Subcutaneous Sensor)	RT‐CGM	isCGM	(1)(8)(9)(10)(11)(12)	Cochrane criteria	GRADE framework
17	2024Uhl[31]	14(1647)	T2D adults (≥18 years)	Continuous Glucose Monitoring System (Subcutaneous Sensor)	CGM	SMBG	(1)(9)(10)(11)	Cochrane criteria	GRADE framework
18	2019Ilaria[32]	12(‐)	T2D	Continuous Glucose Monitoring System (Subcutaneous Sensor)、insulin pump	CGM、FGM	SMBG	(1)(2)(7)(8)(12)(36)(15)	Cochrane criteria	NR
19	2020Maiorino[33]	15(2461)	T1D \T2D	Continuous Glucose Monitoring System (Subcutaneous Sensor)、insulin pump	CGM	usual care	(1)(9)(10)(11)(12)	Cochrane criteria	NR
20	2021Dicembrini[34]	26(2252)	T1D	Continuous Glucose Monitoring System (Subcutaneous Sensor)、subcutaneous insulin pump	CGM	SMBG	(1)(7)(9)(22)(32)(15)	Cochrane criteria	GRADE framework
21	2017Weisman[35]	24(585)	T1D	artificial pancreas (subcutaneous sensor + insulin pump)	artificial pancreas	CSII、SAP	(9)(10)(36)	Cochrane criteria	NR
22	2022Jiao[36]	11(817)	T1D	artificial pancreas (subcutaneous sensor + automated insulin pump)	artificial pancreas	CSII、SAP、MDI、PLGS	(7)(8)(9)(10)(11)(12)(14)(15)(36)(39)(40)	Cochrane criteria	NR
23	2022Fang[37]	12(715)	T1D	artificial pancreas (subcutaneously implanted sensor + automated insulin pump)、SAP)with subcutaneous glucose sensor and subcutaneous insulin infusion pump	artificial pancreas	SAP	(9)(14)(16)(22)(39)(40)	Jadad scale、Cochrane criteria	GRADE framework
24	2023Zeng[38]	25(1345)	T1D outpatients aged ≤25 years	AID system with patch insulin pump or wearable insulin pump	AID	CSII、MDI、SAP	(9)(10)(11)(22)(32)	Cochrane criteria	GRADE framework
25	2021Luo[39]	6(1001)	T2D adults (≥18 years)	patch insulin pump、hip‐worn wearable device、activity tracker、ingestible sensor、wearable sensor patch (for medication adherence tracking)	wearable device	usual care	(1)	Cochrane criteria	NR
26	2022Luo [40]	8(1587)	patients at risk of diabetic foot ulcer	therapeutic footwear	therapeutic offloading footwear	using regular footwear	(33)	Cochrane criteria	GRADE framework
27	2020Alahakoon[41]	17(2729)	patients at risk of diabetic foot ulcer	Infrared thermometer for home foot‐temperature monitoring、custom orthoses for pressure offloading or therapeutic footwear	home foot‐temperature monitoring、offloading shoes	usual care	(33)(37)(38)	Cochrane criteria	NR
28	2014Qiu[42]	11(1423)	T2D	pedometer、accelerometer	PA	usual care	(1)(24)	Cochrane criteria	NR
29	2024de Oliveira[43]	24(1969)	T2D	pedometer、accelerometer	PA	usual care	(1)(8)(17)(18)(19)(20)(21)(23)(24)(26)(27)	Cochrane criteria	GRADE framework
30	2023Zhang[44]	10(1260)	T2D	smart wristband、heart rate armband、muscle oxygen monitor	interventions using exercise management‐integrated wearable devices	standard or other glucose management approaches	(1)(17)(18)(19)(20)(21)	Cochrane criteria	NR
31	2024Chua[45]	19(2547)	T2D adults (≥18 years)	pedometer、accelerometer、clip‐on activity tracker、heart rate strap、wrist‐worn smart band、waist‐worn activity monitor	interventions using exercise management‐integrated wearable devices	usual care	(1)(17)(23)(24)(25)(26)(27)(28)(29)	Cochrane criteria	GRADE framework

Note: Outcomes: (1)glycated Hemoglobin (HbA1c) level；(2)hypoglycemia incidence rate；(3)hyperglycemia incidence rate；(4)amplitude of glycemic fluctuations；(5)diabetic ketoacidosis (DKA) incidence rate；(6)local adverse event incidence rate；(7)quality of life；(8)body weight；(9)time in range(TIR)(3.9–10 mmol/L)；(10)time below range(TBR)(<3.9 mmol/L)；(11)time above range(TAR)(>10 mmol/L)；(12)coefficient of variation of glucose (CV)；(13)incidence of microvascular and macrovascular complications；(14)standard deviation of glucose (SD)；(15)diabetes treatment satisfaction；(16)mean glucose (MG)；(17)fasting plasma glucose (FPG)；(18)total cholesterol (TC)；(19)triglycerides (TG)；(20)low‐density lipoprotein cholesterol (LDL‐C)；(21)high‐density lipoprotein cholesterol(HDL‑C)；(22)number of severe hypoglycemic events；(23)body mass index (BMI)；(24)daily step count；(25)moderate‐to‐vigorous physical activity (MVPA)；(26)systolic blood pressure (SBP)；(27)diastolic blood pressure (DBP)；(28)high‐density lipoprotein (HDL)；(29)low‐density lipoprotein (LDL)；(30)systolic blood pressure / diastolic blood pressure (SBP/DBP)；(31)psychological stress；(32)number of diabetic ketoacidosis (DKA) episodes；(33)diabetic foot ulcer incidence rate；(34)blood lipids；(35)area under the curve (AUC) for hypoglycemia (<3.9 mmol/l)；(36)daily insulin dose；(37)amputation rate (minor, major, total)；(38)adherence to pressure‐relieving footwear；(39)low blood glucose index (LBGI)；(40)high blood glucose index (HBGI).

Abbreviations: Types of wearable devices—AID, automated insulin delivery; CGM, continuous glucose monitoring; CSII, continuous subcutaneous insulin infusion; FGM, flash glucose monitoring; isCGM, intermittently‐scanned continuous glucose monitoring; MDI, multiple daily insulin injection; PA, pedometer/accelerometer‐based physical activity interventions; PLGS, predictive low glucose suspend system; PLGS, predictive low glucose suspend system; RT‐CGM: real‐time continuous glucose monitoring; SAP, sensor‐augmented pump; SMBG, self‐monitoring of blood glucose. Quality assessment—NR, not reported.

### 3.3. Quality Assessment

#### 3.3.1. Report Quality Assessment

None of the included studies fully complied with the PRISMA 2020 reporting standards, with the highest score being 26.5 points [38] and the lowest 20 points [21].

Nineteen out of 27 items have a “yes” response rate of more than 90%, and this shows that the report was relatively complete. However, there were some reporting deficiencies in other items. The reports of Q15 (certainty assessment), Q22 (certainty of evidence), Q24 (registration and protocol), and Q27 (availability of data, code, and other materials) were incomplete (the “yes” response rate was less than 50%). These four under‐reported items are core pillars of methodological quality, and their omission renders the methodological information of some studies difficult or even impossible to verify.

**Figure 2:**
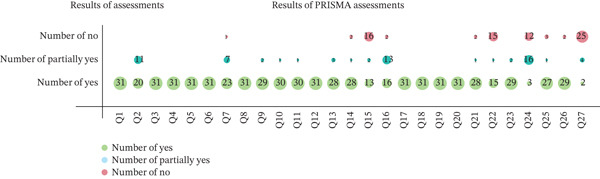
PRISMA 2020 report quality evaluation results.

Particularly, the protocol and registration information were reported in only three studies [37, 41, 42] and the data accessibility was inadequately reported, with only two studies specifying data retrieval pathways [25, 38]. The results of the PRISMA inventory evaluation are shown in Figure 2 and Tables S1 and S2.

#### 3.3.2. Methodological Quality Assessment

**Figure 3:**
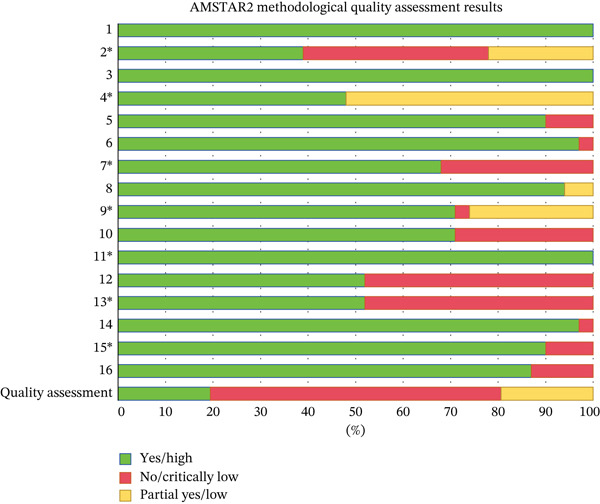
AMSTAR2 methodological quality assessment results.

The AMSTAR‐2 appraisal revealed significant methodological heterogeneity among the reviewed studies. Out of the articles evaluated, six were classified as high quality (19.4%), six as low quality (19.4%), and 19 as very low quality (61.2%), resulting in an overall low‐quality assessment. Methodological quality limitations come from the following items: Item 2 (only 12 studies provided information on trial registration), Item 4 (16 studies employed insufficiently comprehensive search strategies), and Item 13 (15 studies did not discuss the impact of risk of bias on study outcomes). The distribution of methodological quality ratings across items is shown in Figure 3 and Table S3 ( ^∗^ The key items of the AMSTAR‐2).

#### 3.3.3. Evidence Quality Assessment

A total of four categories of outcome measures (glucose‐related indicators, diabetes complication‐related indicators, weight, exercise, and health indicators, psychological state indicators), 97 evidence items, and 27 specific indicators were evaluated. The evidence quality was predominantly low grade: one item (1.03%) was high quality (HbA1c levels, RT‐CGM/isCGM vs. SMBG) [25], 19 (19.59%) were of moderate quality (e.g., TIR, systolic blood pressure, and HbA1c levels), 55 (56.7%) were of low quality (e.g., time below range [TBR] and daily step count), and 22 (22.68%) were of very low quality (e.g., total cholesterol and diastolic blood pressure). GRADE‐specific assessment details are shown in Table 3.

**Table 3:** Result of GRADE assessments.

Outcomes	Study	Interventions versus comparisons	No.RCTs/pts	Effect size (95% CI)	*p*	*I* ^2^%	Certainty assessment					GRADE quality
							Risk of bias	Inconsistency	Indirectness	Imprecision	Publication bias	
	Glucose‐related indicators											
TIR	[22]	CGM versus SMBG	18 (NR)	MD = 5.37[3.54, 7.20]	NR	71.29	−1^a^	−1^b^	0	0	0	Low
	[24]	CGM versus SMBG	11 (1745)	MD = 91.53 [65.77, 117.28]	< 0.001 ^∗^	64	−1^a^	−1^b^	0	0	0	Low
	[25]	RT‐CGM/isCGM versus SMBG	5 (344)	WMD = 8.63[4.54, 12.71]	< 0.01 ^∗^	0	0	0	0	−1^c^	0	Moderate
	[27]	RT‐CGM/isCGM versus SMBG	8 (937)	MD = 6.36 [2.48, 10.24]	0.001 ^∗^	9	−1^a^	0	0	0	−1^f^	Low
	[29]	CGM versus SMBG	6 (410)	MD = 2.51 [−6.08, 11.10]	0.01 ^∗^	75.9	−1^a^	−1^b^	0	0	0	Low
	[30]	RT‐CGM versus isCGM	5 (446)	MD = 7.04[5.76, 8.33]	0.45	0	−1^a^	0	0	0	−1^e^	Low
	[31]	CGM versus SMBG	4 (467)	MD = 11.06[1.80, 20.32]	0.203	34.8	−1^a^	0	0	0	−1^e^	Low
	[33]	CGM versus SMBG	15 (2461)	WMD = 70.74 [46.73,94.76]	0.000 ^∗^	66.3	−1^a^	−1^b^	0	0	0	Low
	[35]	Artificial Pancreas System versus usual care	27 (1170)	MD = 12.59 [9.02, 16.16]	< 0.001 ^∗^	84	−1^a^	−1^b^	0	0	0	Low
	[36]	CLS versus usual care	10 (613)	MD = 10.32 [8.70, 11.95]	< 0.001 ^∗^	21	−1^a^	0	0	0	0	Moderate
	[37]	CGM versus SMBG	7 (1007)	MD = 8.49 [3.96, 13.02]	< 0.001 ^∗^	64	−1^a^	−1^b^	0	0	0	Low
	[37]	CLS versus SAP	11 (622)	MD = 7.91 [4.45, 11.37]	< 0.001 ^∗^	67	−1^a^	−1^b^	0	0	0	Low
	[38]	AID versus usual care	25 (1325)	MD = 11.38 [9.01, 13.76]	< 0.001 ^∗^	88.51	−1^a^	−1^b^	0	0	0	Low
TBR	[20]	RCGM versus SMBG	3 (284)	SMD = −0.35 [−0.59, −0.10]	0.006 ^∗^	0	−1^a^	0	0	−1^c^	−1^e^	Very low
	[22]	CGM versus SMBG	17 (NR)	MD = −1.77 [−2.7, −0.83]	NR	84.08	−1^a^	−1^b^	0	0	0	Low
	[24]	CGM versus SMBG	10 (1629)	MD = −31.98 [−44.11, −19.54]	< 0.001 ^∗^	98	−1^a^	−1^b^	0	0	0	Low
	[25]	RT‐CGM/isCGM versus SMBG	7 (407)	WMD = −0.58 [−1.23,0.08]	0.09	84	0	−1^b^	0	−1^d^	0	Low
	[27]	RT‐CGM/isCGM versus SMBG	9 (956)	MD = −0.66 [−1.21, −0.12]	0.02 ^∗^	45	−1^a^	0	0	0	−1^f^	Low
	[28]	CGM versus SMBG	6 (764)	MD = −0.30 [−0.49, −0.13]	0.001 ^∗^	58	−1^a^	−1^b^	0	0	0	Low
	[30]	RT‐CGM versus isCGM	5 (446)	MD = −1.70 [−2.98, −0.42]	< 0.01 ^∗^	88	−1^a^	−1^b^	0	0	−1^e^	Very low
	[31]	CGM versus SMBG	5 (492)	MD = −0.44 [−1.19,0.31]	0.065	54.8	−1^a^	−1^b^	0	−1^d^	0	Very low
	[35]	Artificial Pancreas System versus usual care	21 (NR)	MD = −2.45 [−3.79, −1.11]	0.0003 ^∗^	94	−1^a^	−1^b^	0	0	0	Low
	[38]	AID versus usual care	24 (1287)	MD = −0.58 [−1.02, −0.15]	0.008 ^∗^	85.98	−1^a^	−1^b^	0	0	0	Low
TAR	[24]	CGM versus SMBG	9 (1388)	MD = −45.1 [−76.34, −13.87]	0.005 ^∗^	68	−1^a^	−1^b^	0	0	0	Low
	[25]	RT‐CGM/isCGM versus SMBG	5 (344)	WMD = −7.75 [−12.04, −3.45]	< 0.01 ^∗^	0	0	0	0	−1^c^	0	Moderate
	[27]	RT‐CGM/isCGM versus SMBG	8 (937)	MD = −5.86 [−10.88, −0.84]	0.02 ^∗^	37	−1^a^	0	0	0	−1^f^	Low
	[28]	CGM versus SMBG	6 (764)	MD = −0.30 [−0.49, −0.13]	0.01 ^∗^	76	−1^a^	−1^b^	0	0	0	Low
	[30]	RT‐CGM versus isCGM	5 (446)	MD = −4.07 [−6.78, −1.36]	0.09	50	−1^a^	0	0	0	−1^e^	Low
	[31]	CGM versus SMBG	4 (467)	MD = −10.37 [−21.55,0.80]	0.117	49.1	−1^a^	0	0	0	−1^e^	Low
	[38]	AID versus usual care	19 (1032)	MD = −12.19 [14.65, −9.73]	< 0.001 ^∗^	NR	−1^a^	−1^b^	0	0	0	Low
HbA1c Level	[15]	CGMS versus SMBG	7 (335)	MD = −0.22 [−0.439,0.004]	0.055	NR	−1^a^	0	0	−1^c^	−1^f^	Very low
	[16]	RT‐CGM versus SMBG	6 (892)	MD = −0.30 [−0.43, −0.17]	0.092	47.2	−1^a^	0	0	0	0	Moderate
	[17]	RT‐CGM versus SMBG	7 (948)	MD = −0.25 [−0.34, −0.16]	< 0.001 ^∗^	0	−1^a^	0	0	0	0	Moderate
	[18]	CGM versus SMBG	4 (228)	MD = −0.31 [−0.6, −0.22]	0.004 ^∗^	0	−1^a^	0	0	−1^c^	−1^ef^	Very low
	[19]	RT‐CGM versus SMBG	9 (1433)	MD = −0.28 [−0.47, −0.09]	0.004 ^∗^	82	−1^a^	−1^b^	0	0	0	Low
	[20]	CGM versus SMBG	7 (660)	SMD = −0.42 [−0.70, −0.13]	0.01 ^∗^	64	−1^a^	−1^b^	0	0	0	Low
	[22]	CGM versus SMBG	26 (NR)	MD = −0.22 [−0.31, −0.13]	NR	79	−1^a^	−1^b^	0	0	0	Low
	[23]	CGM versus SMBG	21 (2149)	MD = −2.46 [−3.83, −1.09]	0.0005 ^∗^	72	−1^a^	−1^b^	0	0	0	Low
	[24]	CGM versus SMBG	11 (1745)	MD = −0.23 [−0.35, −0.11]	< 0.001 ^∗^	70	−1^a^	−1^b^	0	0	0	Low
	[25]	RT‐CGM/isCGM versus SMBG	7 (407)	WMD = −0.31 [−0.42, −0.21]	< 0.01 ^∗^	0	0	0	0	0	0	High
	[26]	CGM versus SMBG	17 (1619)	SMD = −0.37 [−0.63, −0.11]	< 0.001 ^∗^	82.7	−1^a^	−1^b^	0	0	0	Low
	[27]	RT‐CGM versus SMBG	8 (720)	MD = −3.95 [−5.46, −2.44]	< 0.001 ^∗^	0	−1^a^	0	0	0	−1^f^	Low
	[27]	IsCGM versus SMBG	4 (528)	MD = −1.79 [−5.28, 1.69]	0.31	64	−1^a^	−1^b^	0	−1^d^	−1^ef^	Very low
	[28]	CGM versus SMBG	11 (1425)	MD = −0.31 [−0.45, −0.17]	< 0.001 ^∗^	39	−1^a^	0	0	0	0	Moderate
	[28]	RT‐CGM versus SMBG	6 (607)	MD = −0.39 [−0.55, −0.23]	< 0.001 ^∗^	0	−1^a^	0	0	0	0	Moderate
	[29]	CGM versus SMBG	14 (972)	MD = −0.19 [−0.34, −0.04]	0.245	19.1	−1^a^	0	0	0	0	Moderate
	[30]	RT‐CGM versus isCGM	3 (331)	MD = −0.08 [−0.47, 0.31]	< 0.01 ^∗^	92	−1^a^	−1^b^	0	−1^cd^	−1^e^	Very low
	[31]	RT‐CGM versus SMBG	9 (806)	MD = −0.34 [−0.48, −0.20]	0.981	0	−1^a^	0	0	0	0	Moderate
	[31]	FGM versus SMBG	5 (716)	MD = −0.33 [−0.61, −0.05]	0.069	54	−1^a^	−1^b^	0	0	0	Low
	[31]	CGM versus SMBG	14 (1647)	MD = −0.32 [−0.41, −0.23]	0.623	0	−1^a^	0	0	0	0	Moderate
	[32]	CSII versus MDI	5 (679)	MD = −0.26 [−0.74, 0.22]	0.29	90	−1^a^	−1^b^	0	−1^d^	0	Very low
	[32]	CGM versus SMBG	4 (439)	MD = −0.28 [−0.43, −0.13]	0.0002 ^∗^	0	1^a^	0	0	0	−1^e^	Low
	[33]	CGM versus SMBG	15 (2461)	WMD = −0.17 [−0.29, −0.06]	0.000 ^∗^	92.6	−1^a^	−1^b^	0	0	0	Low
	[34]	CGM versus SMBG	23 (2725)	MD = −0.24 [−0.34, −0.14]	< 0.001 ^∗^	54	−1^a^	−1^b^	0	0	0	Low
	[36]	CLS versus usual care	8 (472)	MD = −0.30 [−0.41, −0.19]	< 0.001 ^∗^	0	−1^a^	0	0	0	0	Moderate
	[39]	Wearable devices versus usual care	6 (1001)	WMD = −0.29 [−0.51, −0.07]	0.025 ^∗^	29.2	−1^a^	0	0	0	0	Moderate
	[43]	PA versus usual care	20 (1790)	MD = −0.22 [−0.40, −0.05]	< 0.01 ^∗^	77	−1^a^	−1^b^	0	0	0	Low
	[44]	Exercise management‐integrated wearable devices versus Standard treatment	10 (1260)	SMD = −0.49 [−0.62, −0.36]	< 0.01 ^∗^	79	−1^a^	−1^b^	0	0	0	Low
	[45]	Wearable devices versus usual care	20 (2047)	MD = −0.04 [−0.21, 0.13]	0.6287	54	−1^a^	−1^b^	0	−1^d^	0	Very low
Incidence of hypoglycemia	[17]	RT‐CGM versus SMBG	6 (864)	MD = 0.69 [0.41, 1.14]	0.15	0	−1^a^	0	0	−1^d^	0	Low
	[23]	CGM versus SMBG	14 (1644)	RR = 0.61 [0.33, 1.15]	0.13	50	−1^a^	0	0	−1^d^	0	Low
	[34]	CGM versus SMBG	23 (2302)	MD = 0.53 [0.28, 0.97]	0.04 ^∗^	60	−1^a^	−1^b^	0	0	0	Low
Fasting plasma glucose	[40]	PA versus usual care	12 (747)	MD = −2.53 [−11.57, 6.50]	< 0.01 ^∗^	85	−1^a^	−1^b^	0	−1^d^	0	Very low
	[44]	Exercise management‐integrated wearable devices versus Standard treatment	9 (1193)	SMD = −0.87 [−1.04, −0.7]	< 0.001 ^∗^	46	−1^a^	0	0	0	0	Moderate
	[45]	Wearable devices versus usual care	6 (224)	MD = −7.60 [−27.41, 12.21]	0.37	93	−1^a^	−1^b^	0	−1^d^	0	Very low
Average blood glucose level	[40]	CLS versus SAP	12 (642)	MD = −0.25 [−0.48, −0.08]	0.003 ^∗^	45	−1^a^	0	0	0	0	Moderate
Hypoglycemic index	[40]	CLS versus SAP	7 (396)	MD = −0.41 [−0.55, −0.26]	< 0.001 ^∗^	15	−1^a^	0	0	−1^c^	0	Low
Hyperglycemia index	[40]	CLS versus SAP	5 (322)	MD = −2.56 [−3.38, −1.74]	< 0.001 ^∗^	0	−1^a^	0	0	−1^c^	0	Low
SD	[25]	RT‐CGM/isCGM versus SMBG	3 (247)	WMD = −4.00 [−6.86, −1.14]	< 0.01 ^∗^	0	0	0	0	−1^c^	‐1^e^	Low
	[40]	CLS versus SAP	10 (404)	MD = −0.25 [−0.44, −0.06]	0.01 ^∗^	58	−1^a^	−1^b^	0	0	0	Low
Area under the hypoglycemic curve	[16]	RT‐CGM versus SMBG	6 (892)	MD = −0.26 [−0.64, 0.11]	0.004 ^∗^	71.2	−1^a^	−1^b^	0	−1^d^	0	Very low
Daily insulin dose	[35]	Artificial pancreas system versus conventional pump therapy	18 (NR)	MD = 0.1 [−0.04, 0.16]	0.08	31	−1^a^	0	0	−1^d^	0	Low
	Indicators of Diabetes Complications											
DKA	[23]	CGM versus SMBG	13 (1546)	RR = 1.06 [0.49, 2.32]	0.88	0	−1^a^	0	0	−1^d^	0	Low
	[34]	CGM versus SMBG	23 (1963)	MD = 0.49 [0.23, 1.04]	0.06	0	−1^a^	0	0	0	0	Moderate
Incidence of foot ulcers	[40]	Special therapeutic footwear versus usual care	8 (1587)	RR = 0.49 [0.28, 0.84]	< 0.01 ^∗^	68	−1^a^	−1^b^	0	0	0	Low
	[41]	Home foot temperature monitoring versus usual care	4 (468)	OR = 0.51 [0.31, 0.84]	0.009 ^∗^	0	1^a^	0	0	0	−1^e^	Low
	Weight, exercise and health indicators											
Body weight	[43]	PA versus usual care	15 (1526)	MD = 0.24 [−1.93, 2.40]	< 0.01 ^∗^	53	−1^a^	−1^b^	0	−1^d^	0	Very low
BMI	[43]	PA versus usual care	14 (1248)	MD = −0.31 [−1.00, 0.37]	0.04 ^∗^	42	−1^a^	0	0	−1^d^	0	Low
	[45]	Wearable devices versus usual care	9 (900)	SMD = 0.54 [0.18, 0.90]	0.02 ^∗^	0	−1^a^	0	0	0	0	Moderate
MVPA	[45]	Wearable devices versus usual care	2 (432)	MD = 7.69 [−23.82, 39.20]	0.2	11	−1^a^	0	0	−1^d^	−1^e^	Very low
Daily step count	[42]	PA versus usual care	7 (861)	MD = 1822 [751, 2894]	< 0.001 ^∗^	85.9	−1^a^	−1^b^	0	0	0	Low
	[43]	PA versus usual care	10 (878)	MD = 2131.61 [1348.34, 2914.88]	< 0.01 ^∗^	74	−1^a^	−1^b^	0	0	0	Low
	[45]	Wearable devices versus usual care	11 (1076)	MD = 1583 [447, 2719]	< 0.05 ^∗^	83	−1^a^	−1^b^	0	0	0	Low
Triglycerides	[44]	Exercise management−integrated wearable devices versus standard treatment	7 (1070)	SMD = −0.28 [−0.42, −0.15]	< 0.001 ^∗^	28	−1^a^	0	0	0	0	Moderate
HDL‐C	[44]	Exercise management‐integrated wearable devices versus Standard treatment	7 (1070)	SMD = 0.0 7 [0.01, 0.13]	0.02 ^∗^	20	−1^a^	0	0	0	0	Moderate
Total cholesterol	[43]	PA versus usual care	13 (1485)	MD = 0.42 [−4.57, 1.39]	< 0.01 ^∗^	53	−1^a^	−1^b^	0	−1^d^	0	Very low
	[44]	Exercise management‐integrated wearable devices versus Standard treatment	7 (1070)	SMD = −0.25 [−0.37, 0.12]	0.27	89	−1^a^	−1^b^	0	−1^d^	0	Very low
LDL‐C	[44]	Exercise management‐integrated wearable devices versus Standard treatment	7 (1070)	SMD = −0.12 [−0.19, 0.06]	0.22	91	−1^a^	−1^b^	0	−1^d^	0	Very low
HDL	[29]	CGM versus SMBG	3 (107)	MD = 0.03 [−0.2, 0.27]	0.009 ^∗^	78.9	−1^a^	−1^b^	0	−1^c^	−1^e^	Very low
	[45]	Wearable devices versus usual care	7 (740)	MD = 0.02 [−0.04, 0.07]	0.44	0	−1^a^	0	0	−1^d^	0	Low
LDL	[45]	Wearable devices versus usual care	7 (993)	MD = −0.06 [−0.16, 0.05]	0.24	0	−1^a^	0	0	−1^d^	0	Low
SBP	[43]	PA versus usual care	17(1715)	MD = 1.37[−1.38, 4.12]	< 0.01 ^∗^	79	−1^a^	−1^b^	0	−1^d^	0	Very low
	[45]	Wearable devices versus usual care	11 (1158)	MD = −2.46 [−4.13, −0.79]	< 0.01 ^∗^	0	−1^a^	0	0	0	0	Moderate
DBP	[43]	PA versus usual care	16 (1623)	MD = 0.34 [−1.40, 2.07]	< 0.01 ^∗^	82	−1^a^	−1^b^	0	−1^d^	0	Very low
	[45]	Wearable devices versus usual care	11 (1158)	MD = −1.00 [−3.95, 1.96]	0.47	75	−1^a^	−1^b^	0	−1^d^	0	Very low
	Indicators of psychological state											
Treatment satisfaction	[25]	RT‐CGM/isCGM versus SMBG	4 (267)	SMD = 0.79 [0.54, 1.05]	< 0.01 ^∗^	0	0	0	0	−1^c^	−1^e^	Low
	[29]	CGM versus SMBG	3 (96)	MD = −0.54 [−0.98, −0.11]	NR	NR	−1^a^	−1^b^	0	−1^c^	−1^e^	Very low

*Note:* −1—Downgrade by one level; 0—not downgrade.

Abbreviations: AID, automated insulin delivery; BMI, body mass index; CGM, continuous glucose monitoring; CGMS, continuous glucose monitoring system; CI, confidence interval; CLS, closed‐loop system; CSII, continuous subcutaneous insulin infusion; CV, coefficient of variation of glucose; DBP, diastolic blood pressure; DKA, diabetic ketoacidosis incidence rate; FGM, flash glucose monitoring; HbA1c, glycated hemoglobin; HDL, high‐density lipoprotein; HDL‐C, high‐density lipoprotein cholesterol; isCGM, intermittently‐scanned continuous glucose monitoring; LDL, low‐density lipoprotein; LDL‐C, low‐density lipoprotein cholesterol; MD, mean difference; NR, not report; MDI, multiple daily insulin injection; MVPA, moderate‐to‐vigorous physical activity; OR, odds ratio; PA, pedometer/accelerometer‐based physical activity interventions; rCGM, retrospective continuous glucose monitoring; RR, risk ratio; RT‐CGM, real‐time continuous glucose monitoring; SAP, sensor‐augmented pump; SBP, systolic blood pressure; SMBG, self‐monitoring of blood glucose; SMD, standardized mean difference; TAR, time above range (> 10 mmol/L); TBR, time below range (<3.9 mmol/L); TIR, time in range (3.9–10 mmol/L); WMD, weighted mean difference.

^a^The included studies have a large bias in methodology such as randomization, allocation concealment, and blinding.

^b^Small overlap of confidence intervals between studies, large *I* ^2^ values, and large heterogeneity. *I* ^2^ value greater than 50% indicates substantial heterogeneity. The high *I* ^2^ values were primarily related to factors such as the type of CGM, characteristics of the study population (age, baseline HbA1c, disease duration, BMI, and insulin therapy status), study design and methodology (intervention duration, number of study centers, and completeness of randomization and allocation concealment), outcome measurement criteria (TIR glucose range, definition of hypoglycemia, and whether data were presented as mean or median), as well as the insulin treatment regimen (MDI, CSII, or hybrid protocols).

^c^Does not meet the optimal informative sample size (sample size needs to be greater than 400).

^d^Confidence intervals cross the null line.

^e^Small number of included studies and possible publication bias.

^f^Funnel plot asymmetry.

^∗^Represented a significant difference between intervention and control group, *p* < 0.05.

### 3.4. Types and Characteristics of Wearable Devices

The wearable devices included in the study were categorized into four types, with blood glucose monitoring applications being the most prevalent (21/31, 67.7%): ?blood glucose monitoring—primarily CGM systems, including real‐time CGM (RT‐CGM) and intermittent scanning CGM (isCGM). Their core advantage lies in overcoming the limitations of fingerstick blood sampling to detect hidden fluctuations such as nocturnal hypoglycemia and postprandial hyperglycemia. ? Therapeutic devices—comprising insulin pumps (e.g., patch‐type, and wearable), specialized therapeutic footwear, and foot infrared thermometers, enabling precise insulin delivery regulation or reduced risk of foot ulcers. ? Exercise intervention devices—such as pedometers and smart wristbands, quantifying steps, heart rate, and other data to assist in developing exercise programs. ? Integrated management solutions—such as artificial pancreas systems (closed − loop CGM + insulin pump systems) and medication reminder patches, enabling seamless integration of monitoring‐intervention‐recording.

### 3.5. Results of the Systematic Evaluation of Outcome Indicators

#### 3.5.1. Glucose‐Related Indicators

**Figure 4:**
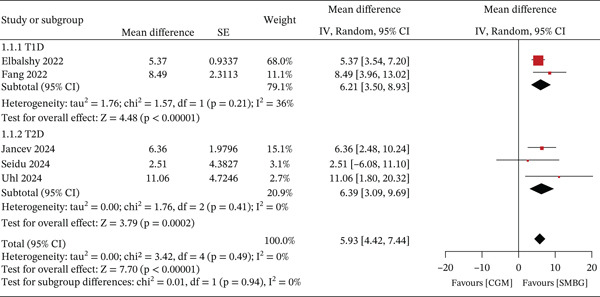
Meta‐analysis of TIR.

Twelve reviews evaluated the effect of wearable devices on time in range (TIR), with 10 showing significantly higher TIR in the intervention group (*p* < 0.05). Two reviews of moderate‐quality evidence indicate that compared with SMBG, the use of RT‐CGM/isCGM significantly improves TIR levels [25]; and that closed‐loop systems demonstrate greater improvement in TIR levels compared with usual care [36]. Six studies included in this review reported the effects of CGM on TIR in patients with diabetes compared with SMBG. Of these, one study enrolled mixed populations of patients with T1D and T2D, whereas two studies focused exclusively on T1D and three on T2D. Quantitative analysis using a random‐effects model demonstrated that CGM was associated with a statistically significant improvement in TIR compared with SMBG (MD = 5.93, 95% CI [4.42, 7.44], *p* < 0.01), without significant heterogeneity (*I* ^2^ = 0%). Subgroup analyses showed that CGM significantly improved TIR in patients with T1D (MD = 6.21, 95% CI [3.50, 8.93]) and in those with T2D (MD = 6.39, 95% CI [3.09, 9.69]) compared with SMBG. However, no statistically significant difference was observed between the two subgroups (*p* > 0.05). Details are presented in Figure 4.

Twenty‐two reviews assessed the impact of wearable devices on HbA1c levels, with 18 reporting significant reductions in the intervention group (*p* < 0.05). RT‐CGM showed superior efficacy to isCGM, whereas CGM overall outperformed SMBG. One study [25] had high‐level evidence, four [17, 28, 31, 44] had moderate‐level evidence, and the remainder were mostly low or very low‐level evidence.

**Figure 5:**
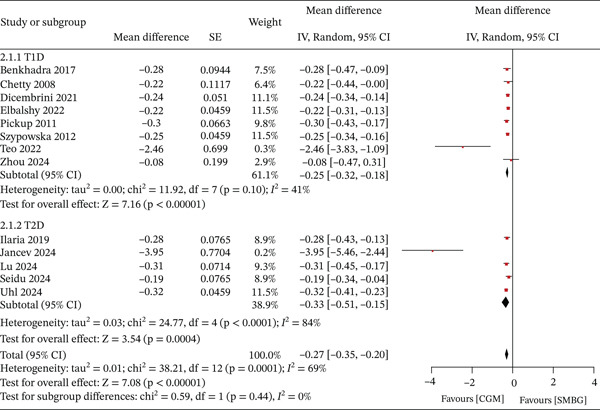
Meta‐analysis of HbA1c.

Fifteen studies included in this review reported the effects of CGM on HbA1c in patients with diabetes compared with SMBG. Of these, two studies enrolled mixed populations of patients with T1D and T2D, whereas eight studies focused exclusively on T1D and five on T2D. Quantitative analysis using a random‐effects model demonstrated that CGM was associated with a statistically significant improvement in HbA1c compared with SMBG (MD = −0.27, 95% CI [−0.35, −0.20], *p* < 0.01), with substantial heterogeneity observed among studies (*I* ^2^ = 69%). Subgroup analyses showed that CGM significantly reduced HbA1c in patients with T1D (MD = −0.25, 95% CI [−0.32, −0.18]) and in those with T2D (MD = −0.33, 95% CI [−0.51, −0.15]) compared with SMBG. However, no statistically significant difference was observed between the two subgroups (*p* > 0.05). Details are presented in Figure 5.

Nine reviews reported TBR, with seven showing reductions in the intervention group. Seven reviews reported TAR, with six showing TAR reduction in the intervention group. Only one [25] had moderate‐level evidence for TAR, with the remainder being low or very low quality.

#### 3.5.2. Indicators of Diabetes Complications

Two reviews [23, 34] evaluated the effect of CGM versus SMBG on DKA risk, with no statistically significant differences observed between intervention and control groups. Two reviews [40, 41] demonstrated that therapeutic offloading footwear and home foot‐temperature monitoring significantly reduced foot ulcer risk, with evidence quality rated as low in both cases.

#### 3.5.3. Weight, Exercise, and Health Indicators

Two reviews [43, 45] indicated that wearable device‐related interventions improved BMI (moderate‐quality evidence) but had no significant effect on body weight (low‐quality evidence). Three reviews evaluated the effect of wearable activity trackers on daily step counts in patients with T2D [42, 43, 45], all reporting statistically significant increases in the intervention group (*p* < 0.05) but with low evidence certainty. Four reviews [29, 43–45] reported improvements in cardiovascular metabolic indicators with wearable device‐based interventions: One [44] demonstrated significant improvements in triglycerides and high‐density lipoprotein cholesterol, and one [45] reported reduced systolic blood pressure (both moderate‐quality evidence).

#### 3.5.4. Indicators of Psychological State

Two studies [25, 29] reported patient satisfaction. One [25] (RT‐CGM/isCGM versus SMBG) demonstrated higher satisfaction in the intervention group. The outcome of the other [29] was inconclusive. The evidence is of low or very low quality, lacking replication.

## 4. Discussion

### 4.1. Current Status and Effectiveness of Wearable Device–Based Interventions in Diabetes Management

Consistent with previous findings, existing evidence indicates that wearable device–based interventions demonstrate clear efficacy in core aspects of diabetes management. Regarding glycemic control, CGM and closed‐loop insulin delivery systems (AID, CLS, and artificial pancreas) have been shown to significantly improve TIR, reduce HbA1c levels, and decrease TBR/time above range (TAR), thereby enhancing glycemic stability in both Type 1 and T2D patients [24, 25, 35, 36]. These effects are primarily attributable to the real‐time glucose feedback of CGM and the automated insulin adjustments of closed‐loop systems [46].

In exercise management, pedometers and smart wristbands effectively increase daily step counts and physical activity levels, contributing to improvements in lipid profiles and blood pressure [42–45]. This observation aligns with established evidence that exercise interventions enhance insulin sensitivity [47], suggesting that wearable devices can facilitate adherence to exercise prescriptions.

Regarding complication prevention, therapeutic footwear and foot temperature monitoring devices have been shown to significantly reduce the incidence of diabetic foot ulcers [40, 41], addressing limitations in conventional foot care management and highlighting the complementary value of wearable devices.

However, gaps remain in current evidence. Regarding DKA prevention, no significant differences have been observed between intervention and control groups [23, 34], and the available evidence on DKA prevention is limited. This may be attributed to the heterogeneity of study populations, insufficient sample sizes and follow‐up durations, and the fact that current CGM devices primarily focus on real‐time glucose monitoring rather than addressing the underlying causes of DKA, which limits their preventive effectiveness. Similarly, evidence on psychological well‐being, patient satisfaction, and broader outcomes such as quality of life remains limited in both quantity and quality, potentially due to the lack of systematic evaluation of factors such as device comfort and the complexity of data interpretation.

### 4.2. Methodological and Evidence Quality of Included Studies

The included studies generally exhibited low methodological quality and low certainty of evidence, limiting the generalizability of findings. Approximately 80% of studies were downgraded due to insufficient reporting of randomization, allocation concealment, or blinding, potentially introducing subjective bias. Some studies failed to justify sample size calculations, further undermining result credibility [30, 31]. Notably, deficiencies in reporting quality were closely related to methodological limitations. The absence of protocol registration precludes confirmation of the study design′s prospectivity and hinders the rational justification of deviations from the protocol. Likewise, missing descriptions of certainty assessment methods and corresponding results prevented verification of the appropriateness of evidence grading, thereby compromising methodological transparency.

Regarding evidence grading, 76.3% of evidence bodies were rated as low or very low quality (GRADE classification), primarily due to: ? substantial heterogeneity [24, 38], potentially arising from differences in device brands and intervention durations (1–12 months); ? inadequate sample sizes, with most studies including fewer than 400 participants [15, 25, 30]; ? publication bias, as some systematic reviews were downgraded due to limited literature inclusion or asymmetric funnel plots [27, 30, 31]. Inconsistencies in outcome measures and follow‐up durations further complicated interpretation. Although TIR had a standardized definition (3.9–10 mmol/L), monitoring frequency was not consistently reported across studies. Such inconsistencies undermine the reliability of cross‐study comparisons and meta‐analyses.

### 4.3. Implications for Practice and Research

In nursing practice, the application of wearable devices should be tailored according to patient characteristics. For individuals with Type 1 diabetes, RT‐CGM combined with an artificial pancreas system constitutes the optimal approach, as RT‐CGM outperforms isCGM in reducing HbA1c and minimizing hypoglycemic episodes [30], and artificial pancreas systems further enhance TIR [35]. For T2D patients not on insulin therapy, isCGM offers convenience while meeting glycemic monitoring needs [25], with potential long‐term cost benefits [48].

Among high‐risk populations for diabetic foot complications, therapeutic footwear and foot temperature monitoring devices reduce ulcer risk by 51% and 49%, respectively [40, 41], warranting broader adoption. In exercise management, wearable‐based exercise management interventions (e.g., pedometers with daily step targets) effectively improve physical activity and lipid profiles [42, 43, 45].

To optimize nursing practice, future studies should adhere to PRISMA 2020 guidelines, clearly report randomization, blinding, and conflicts of interest, and standardize core outcome measures such as HbA1c (6‐month follow‐up), CGM coverage ≥ 70% [49], and foot ulcer incidence (1‐year follow‐up). Extending intervention durations is essential to evaluate long‐term efficacy and microvascular complications. Research should also assess cost‐effectiveness, nursing staff satisfaction, and adverse skin reactions to fully validate wearable devices′ utility in nursing care.

### 4.4. Limitations

This study has several limitations. First, only Chinese and English literature were included, introducing potential language bias. The included studies used different assessment tools for some outcomes, and some did not report the tools used. Furthermore, this study focused on patient satisfaction and psychological outcomes without assessing the experiences and satisfaction of clinical healthcare providers, who play a key role in monitoring wearable device data.

## 5. Conclusion

Wearable devices are beneficial for diabetes management, with CGM showing the strongest evidence for glycemic control. Activity trackers improve daily physical activity, and foot protection devices reduce the risk of diabetic foot complications. However, evidence for DKA prevention and quality‐of‐life outcomes remains limited, and the included studies generally exhibit methodological weaknesses and low‐quality evidence. Therefore, clinicians should interpret these findings cautiously. Future research should prioritize high‐quality studies with follow‐up durations exceeding 1 year, rigorous methodological designs, standardized outcome measures, and high levels of data transparency to strengthen the evidence base.

## Author Contributions

M.T. and X.L. participated in conceptualizing and designing the study. M.T., M.Z., N.A., and Y.W. conducted a literature search and screened data extraction. M.Z. and Y.W. analyzed the data and did a statistical analysis. M.T. and N.A. wrote a manuscript. The manuscript was revised by X.L.

## Funding

This study was supported by Shanghai Nursing Association Research Project (2025MS‐B04).

## Disclosure

All authors read and approved the final version of the manuscript, and all authors agree to be accountable for the content and conclusions of the article.

## Conflicts of Interest

The authors declare no conflicts of interest.

## Supporting information


**Supporting Information** Additional supporting information can be found online in the Supporting Information section. Table S1: Compliance of PRISMA assessments. Table S2: Result of PRISMA assessments. Table S3: Result of the AMSTAR‐2 assessments.

## Data Availability

The datasets analyzed during the current study are available from the corresponding author on reasonable request.
