# Supplementary material for: Effectiveness of Wearable Devices for Diabetes Management: An Overview of Systematic Reviews and Meta‐Analyses
Source: J Diabetes Res. 2026 Apr 9;2026:1464982. doi: 10.1155/jdr/1464982 (PMC13066504; doi:10.1155/jdr/1464982)
Supplement: Supplementary file 1 — Supporting Information Additional supporting information can be found online in the Supporting Information section. Table S1: Compliance of PRISMA assessments. Table S2: Result of PRISMA assessments. Table S3: Result of the AMSTAR‐2 assessments. [file JDR-2026-1464982-s001.docx]

Supplementary Materials:

Table S1 :Compliance of PRISMA assessments

| Section/topic | Items | Number of yes(%) | Number of partially yes(%) | Number of no(%) |
| --- | --- | --- | --- | --- |
| Title | Q1 Title | 31(100%) | 0(0%) | 0(0%) |
| Abstract | Q2 Abstract | 20(64.5%) | 11(35.5%) | 0(0%) |
| Introduction | Q3 Rationale | 31(100%) | 0(0%) | 0(0%) |
|  | Q4 Objectives | 31(100%) | 0(0%) | 0(0%) |
| Methods | Q5 Eligibility criteria | 31(100%) | 0(0%) | 0(0%) |
|  | Q6 Information sources | 31(100%) | 0(0%) | 0(0%) |
|  | Q7 Search strategy | 23(74.2%) | 7(22.6%) | 1(3.2%) |
|  | Q8 Selection process | 31(100%) | 0(0%) | 0(0%) |
|  | Q9 Data collection process | 29(93.5%) | 2(6.5%) | 0(0%) |
|  | Q10 Data items | 30(96.8%) | 1(3.2%) | 0(0%) |
|  | Q11 Study risk of bias assessment | 30(96.8%) | 1(3.2%) | 0(0%) |
|  | Q12 Effect measures | 31(100%) | 0(0%) | 0(0%) |
|  | Q13 Synthesis methods | 28(90.3%) | 3(9.7%) | 0(0%) |
|  | Q14 Reporting bias assessment | 28(90.3%) | 1(3.2%) | 2(6.5%) |
|  | Q15 Certainty assessment | 13(41.9%) | 2(6.5%) | 16(51.6%) |
| Results | Q16 Study selection | 16(51.6%) | 13(41.9%) | 2(6.5%) |
|  | Q17 Study characteristics | 31(100%) | 0(0%) | 0(0%) |
|  | Q18 Risk of bias in studies | 31(100%) | 0(0%) | 0(0%) |
|  | Q19 Results of individual studies | 31(100%) | 0(0%) | 0(0%) |
|  | Q20 Results of syntheses | 31(100%) | 0(0%) | 0(0%) |
|  | Q21 Reporting biases | 28(90.3%) | 1(3.2%) | 2(6.5%) |
|  | Q22 Certainty of evidence | 15(48.4%) | 1(3.2%) | 15(48.4%) |
| Discussion | Q23 Discussion | 29(93.5%) | 2(6.5%) | 0(0%) |
| Other information | Q24 Registration and protocol | 3(9.7%) | 16(51.6%) | 12(38.7%) |
|  | Q25 Support | 27(87.1%) | 1(3.2%) | 3(9.7%) |
|  | Q26 Competing interests | 29(93.5%) | 0(0%) | 2(6.5%) |
|  | Q27 Availability of data, code and other materials | 2(6.5%) | 4(12.9%) | 25(80.6%) |

Table S2 : Result of PRISMA assessments

| Items | [15] | [16] | [17] | [18] | [19] | [20] | [21] | [22] | [23] | [24] | [25] | [26] | [27] | [28] | [29] | [30] | [31] | [32] | [33] | [34] | [35] | [36] | [37] | [38] | [39] | [40] | [41] | [42] | [43] | [44] | [45] |
| --- | --- | --- | --- | --- | --- | --- | --- | --- | --- | --- | --- | --- | --- | --- | --- | --- | --- | --- | --- | --- | --- | --- | --- | --- | --- | --- | --- | --- | --- | --- | --- |
| Q1 Title | Y | Y | Y | Y | Y | Y | Y | Y | Y | Y | Y | Y | Y | Y | Y | Y | Y | Y | Y | Y | Y | Y | Y | Y | Y | Y | Y | Y | Y | Y | Y |
| Q2 Abstract | Y | Y | Y | Y | PY | PY | PY | PY | Y | Y | Y | Y | Y | PY | Y | Y | Y | PY | Y | PY | Y | Y | Y | Y | Y | PY | PY | PY | Y | Y | PY |
| Q3 Rationale | Y | Y | Y | Y | Y | Y | Y | Y | Y | Y | Y | Y | Y | Y | Y | Y | Y | Y | Y | Y | Y | Y | Y | Y | Y | Y | Y | Y | Y | Y | Y |
| Q4 Objectives | Y | Y | Y | Y | Y | Y | Y | Y | Y | Y | Y | Y | Y | Y | Y | Y | Y | Y | Y | Y | Y | Y | Y | Y | Y | Y | Y | Y | Y | Y | Y |
| Q5 Eligibility criteria | Y | Y | Y | Y | Y | Y | Y | Y | Y | Y | Y | Y | Y | Y | Y | Y | Y | Y | Y | Y | Y | Y | Y | Y | Y | Y | Y | Y | Y | Y | Y |
| Q6 Information sources | Y | Y | Y | Y | Y | Y | Y | Y | Y | Y | Y | Y | Y | Y | Y | Y | Y | Y | Y | Y | Y | Y | Y | Y | Y | Y | Y | Y | Y | Y | Y |
| Q7 Search strategy | Y | Y | Y | Y | Y | Y | Y | N | PY | Y | Y | PY | Y | Y | Y | Y | Y | PY | PY | Y | Y | Y | PY | Y | Y | PY | PY | PY | Y | Y | Y |
| Q8 Selection process | Y | Y | Y | Y | Y | Y | Y | Y | Y | Y | Y | Y | Y | Y | Y | Y | Y | Y | Y | Y | Y | Y | Y | Y | Y | Y | Y | Y | Y | Y | Y |
| Q9 Data collection process | Y | Y | Y | Y | Y | Y | Y | Y | Y | Y | Y | Y | Y | Y | Y | Y | Y | PY | Y | Y | Y | Y | Y | Y | Y | PY | PY | Y | Y | Y | Y |
| Q10 Data items | Y | Y | Y | Y | Y | Y | Y | Y | Y | Y | Y | Y | Y | Y | Y | Y | Y | Y | Y | PY | Y | Y | Y | Y | Y | Y | Y | Y | Y | Y | Y |
| Q11 Study risk of bias assessment | Y | PY | Y | Y | Y | Y | Y | Y | Y | Y | Y | Y | Y | Y | Y | Y | Y | Y | Y | Y | Y | Y | Y | Y | Y | Y | Y | Y | Y | Y | Y |
| Q12 Effect measures | Y | Y | Y | Y | Y | Y | Y | Y | Y | Y | Y | Y | Y | Y | Y | Y | Y | Y | Y | Y | Y | Y | Y | Y | Y | Y | Y | Y | Y | Y | Y |
| Q13 Synthesis methods | Y | Y | Y | Y | Y | Y | Y | Y | Y | Y | Y | Y | Y | Y | Y | Y | Y | Y | Y | PY | Y | Y | Y | Y | Y | Y | PY | PY | Y | Y | Y |
| Q14 Reporting bias assessment | Y | Y | Y | Y | N | Y | N | Y | Y | Y | Y | Y | Y | Y | Y | Y | Y | Y | Y | PY | Y | Y | Y | Y | Y | Y | Y | Y | Y | Y | Y |
| Q15 Certainty assessment | N | N | N | N | N | N | N | N | Y | N | Y | Y | Y | N | Y | Y | Y | PY | Y | Y | N | N | PY | Y | N | PY | N | N | Y | Y | Y |
| Q16 Study selection | Y | N | Y | Y | PY | PY | PY | Y | PY | Y | PY | Y | PY | Y | Y | PY | PY | Y | Y | Y | Y | PY | N | Y | Y | Y | Y | Y | PY | PY | Y |
| Q17 Study characteristics | Y | Y | Y | Y | Y | Y | Y | Y | Y | Y | Y | Y | Y | Y | Y | Y | Y | Y | Y | Y | Y | Y | Y | Y | Y | Y | Y | Y | Y | Y | Y |
| Q18 Risk of bias in studies | Y | Y | Y | Y | Y | Y | Y | Y | Y | Y | Y | Y | Y | Y | Y | Y | Y | Y | Y | Y | Y | Y | Y | Y | Y | Y | Y | Y | Y | Y | Y |
| Q19 Results of individual studies | Y | Y | Y | Y | Y | Y | Y | Y | Y | Y | Y | Y | Y | Y | Y | Y | Y | Y | Y | Y | Y | Y | Y | Y | Y | Y | Y | Y | Y | Y | Y |
| Q20 Results of syntheses | Y | Y | Y | Y | Y | Y | Y | Y | Y | Y | Y | Y | Y | Y | Y | Y | Y | Y | Y | Y | Y | Y | Y | Y | Y | Y | Y | Y | Y | Y | Y |
| Q21 Reporting biases | Y | Y | Y | Y | Y | N | N | Y | Y | Y | Y | Y | Y | Y | Y | Y | Y | Y | Y | PY | Y | Y | Y | Y | Y | Y | Y | Y | Y | Y | Y |
| Q22 Certainty of evidence | N | Y | N | N | N | N | N | N | Y | N | Y | Y | Y | N | Y | Y | Y | PY | Y | Y | N | N | Y | Y | N | PY | N | N | Y | Y | Y |
| Q23 Discussion | Y | Y | Y | Y | Y | Y | Y | Y | Y | PY | Y | Y | Y | Y | Y | Y | Y | Y | Y | Y | Y | Y | Y | Y | Y | Y | Y | PY | Y | Y | Y |
| Q24 Registration and protocol | N | N | N | N | N | N | N | PY | PY | PY | PY | PY | PY | PY | PY | PY | N | PY | N | N | PY | N | Y | PY | N | PY | Y | Y | PY | PY | PY |
| Q25 Support | N | PY | Y | N | Y | Y | Y | Y | Y | Y | Y | Y | Y | Y | Y | Y | Y | Y | Y | Y | Y | Y | Y | Y | Y | Y | Y | Y | Y | N | Y |
| Q26 Competing interests | Y | Y | Y | Y | Y | Y | Y | Y | Y | N | Y | Y | Y | Y | Y | Y | Y | Y | Y | Y | Y | Y | Y | Y | Y | Y | Y | Y | Y | Y | Y |
| Q27 Availability of data, code and other materials | N | N | N | N | N | N | N | N | N | N | Y | N | N | N | N | N | N | PY | N | N | N | N | PY | Y | N | PY | PY | PY | N | N | N |

Legend:N,no;PY,partial yes;Y,yes

Table S3: Result of the AMSTAR-2 assessments

| Citation | 1 | 2* | 3 | 4* | 5 | 6 | 7* | 8 | 9* | 10 | 11* | 12 | 13* | 14 | 15* | 16 | Overall quality |
| --- | --- | --- | --- | --- | --- | --- | --- | --- | --- | --- | --- | --- | --- | --- | --- | --- | --- |
| 2008Chetty^[15]^ | Y | N | Y | PY | Y | Y | N | Y | PY | Y | Y | N | N | Y | Y | Y | critically low |
| 2011 Pickup^[16]^ | Y | N | Y | PY | N | N | N | PY | N | N | Y | N | N | N | N | N | critically low |
| 2012Szypowska^[17]^ | Y | N | Y | PY | Y | Y | Y | Y | Y | N | Y | N | N | Y | N | Y | critically low |
| 2013 Poolsup^[18]^ | Y | N | Y | Y | Y | Y | Y | Y | Y | Y | Y | Y | Y | Y | Y | Y | low |
| 2017 Benkhadra ^[19]^ | Y | Y | Y | Y | Y | Y | N | Y | Y | Y | Y | Y | Y | Y | Y | Y | low |
| 2019 Ida^[20]^ | Y | N | Y | PY | Y | Y | Y | Y | Y | Y | Y | Y | Y | Y | Y | Y | critically low |
| 2020 Cowart^[21]^ | Y | N | Y | PY | Y | Y | Y | Y | Y | Y | Y | Y | Y | Y | Y | Y | critically low |
| 2022 Elbalshy^[22]^ | Y | PY | Y | PY | Y | Y | Y | Y | Y | Y | Y | N | N | Y | Y | Y | critically low |
| 2022 Teo^[23]^ | Y | Y | Y | Y | Y | Y | Y | PY | Y | Y | Y | Y | Y | Y | Y | Y | high |
| 2022Wang^[24]^ | Y | N | Y | PY | Y | Y | N | Y | PY | N | Y | N | N | Y | Y | N | critically low |
| 2024Ferreira^[25]^ | Y | PY | Y | PY | Y | Y | Y | Y | Y | Y | Y | N | N | Y | Y | Y | critically low |
| 2024Kong^[26]^ | Y | Y | Y | Y | Y | Y | Y | Y | Y | Y | Y | Y | Y | Y | Y | Y | high |
| 2024 Jancev^[27]^ | Y | PY | Y | PY | Y | Y | Y | Y | Y | Y | Y | N | N | Y | Y | Y | critically low |
| 2024Lu^[28]^ | Y | Y | Y | Y | Y | Y | Y | Y | Y | Y | Y | Y | Y | Y | Y | Y | high |
| 2024 Seidu^[29]^ | Y | PY | Y | Y | Y | Y | N | Y | Y | Y | Y | Y | Y | Y | Y | Y | critically low |
| 2024 Zhou^[30]^ | Y | PY | Y | PY | Y | Y | Y | Y | Y | Y | Y | N | N | Y | N | Y | critically low |
| 2024 Uhl^[31]^ | Y | Y | Y | Y | Y | Y | Y | Y | PY | Y | Y | Y | Y | Y | Y | Y | low |
| 2019 Ilaria^[32]^ | Y | N | Y | PY | N | Y | N | Y | PY | N | Y | N | N | Y | Y | N | critically low |
| 2020 Maiorino^[33]^ | Y | Y | Y | PY | Y | Y | Y | Y | Y | Y | Y | Y | Y | Y | Y | Y | low |
| 2021Dicembrini^[34]^ | Y | Y | Y | Y | Y | Y | Y | Y | Y | Y | Y | Y | Y | Y | Y | Y | high |
| 2017 Weisman^[35]^ | Y | PY | Y | PY | Y | Y | Y | Y | Y | N | Y | N | N | Y | Y | Y | critically low |
| 2022 Jiao^[36]^ | Y | N | Y | PY | Y | Y | Y | Y | Y | N | Y | N | N | Y | Y | Y | critically low |
| 2022 Fang^[37]^ | Y | Y | Y | Y | Y | Y | N | Y | Y | Y | Y | Y | Y | Y | Y | Y | low |
| 2023Zeng^[38]^ | Y | Y | Y | Y | Y | Y | Y | Y | Y | N | Y | Y | Y | Y | Y | Y | high |
| 2022Luo J^[39]^ | Y | N | Y | PY | Y | Y | Y | Y | PY | Y | Y | N | N | Y | Y | Y | critically low |
| 2022Luo B^[40]^ | Y | N | Y | Y | Y | Y | N | Y | Y | N | Y | Y | Y | Y | Y | Y | critically low |
| 2020 Alahakoon^[41]^ | Y | PY | Y | PY | N | Y | N | Y | PY | N | Y | N | N | Y | Y | N | critically low |
| 2014Qiu^[42]^ | Y | Y | Y | Y | Y | Y | Y | Y | Y | Y | Y | Y | Y | Y | Y | Y | high |
| 2024 de Oliveira^[43]^ | Y | Y | Y | Y | Y | Y | N | Y | Y | Y | Y | Y | Y | Y | Y | Y | low |
| 2023Zang^[44]^ | Y | N | Y | Y | Y | Y | Y | Y | PY | Y | Y | N | N | Y | Y | Y | critically low |
| 2024Chua^[45]^ | Y | Y | Y | Y | Y | Y | Y | Y | PY | Y | Y | N | N | Y | Y | Y | critically low |

Legend: * The key items of the AMSTAR-2;

N: no; PY: partial yes; Y: yes.

Q1. Research questions and criteria included PICO;

Q2*. Published a review protocol prior;

Q3. Explained study design for inclusion criteria;

Q4*. Comprehensive literature search strategy;

Q5. Performed study selection in duplicate;

Q6. Performed data extraction in duplicate;

Q7*. Excluded studies listed and justified;

Q8. Included studies described in adequate detail;

Q9*. Used a satisfactory technique for assessing the risk of bias;

Q10. Reported sources of funding for studies in the review;

Q11*. Used appropriate statistical methods for data synthesis;

Q12. Assessed potential impact of risk of bias in each study;

Q13*.Accounted for risk of bias when interpreting results;

Q14. Observed heterogeneity & impact explained;

Q15*. Investigated publication bias;

Q16. Reported own conflict of interests and funding.
High: No or one non-critical weakness.

Moderate: More than one non-critical weakness.

Low: One critical flaw with or without non-critical weaknesses.

Critically low: More than one critical flaw with or without non-critical weaknesses
